# A rare case of spontaneous bladder wall abscess mimicking bladder tumour in young women

**DOI:** 10.1186/s12894-024-01497-6

**Published:** 2024-05-18

**Authors:** Zico Yusuf Alfarizi, Eko Subekti, Danarto, Prahara Yuri

**Affiliations:** 1 Urology Division, Department of Surgery, Faculty of Medicine, Public Health, and Nursing, Universitas Gadjah Mada / Dr. Sardjito Hospital, Jl. Kesehatan No.1, Yogyakarta, 55281 Indonesia; 2 Urology Division, Department of Surgery, Regional General Banyumas Hospital, Jl. Rumah Sakit No.1, Karangpucung, Kejawar, Kec. Banyumas, Kabupaten Banyumas, Jawa Tengah 53192 Indonesia

**Keywords:** Case report, Bladder wall abscess, Bladder abscess, Benign bladder tumour

## Abstract

**Introduction:**

Abscess of the bladder wall is a rare urological disorder, with a few cases recorded in the literature. The finding of a bladder wall mass via computed tomography (CT) imaging in a visiting patient is the subject of this report.

**Case discussion:**

A 37-year-old woman with persistent pain in the suprapubic area and lower urinary tract symptoms was examined as a case study. Through a CT scan revealed an inhomogeneous structure in the anteroinferior part of the right bladder. A cystoscopy procedure followed by transurethral resection was performed to remove the mass, which was found to be an abscess. A Foley catheter with irrigation was administered after surgery, and the patient goes home in three days.

**Conclusion:**

the patient had no symptoms or discomfort in the lower urinary tract after follow-up. Despite the rarity of bladder wall abscesses, cystoscopy can be used to aid diagnosis. Transurethral resection of bladder wall can reduce the mass and eliminate the possibility of malignancy.

## Introduction

Abscess in the bladder wall is an extremely uncommon condition. Three reports have been found in the current literature regarding bladder wall abcsess [1–3].

The etiology, epidemiology, clinical manifestations, and management of bladder wall abscesses remain unclear currently owing to the lack of literature on the subject. A comprehensive examination of the relevant literature indicates that the occurrence rate of abscesses in the urachus and intramural space is marginally greater than that of abcesses that affect the bladder wall. Therefore, we present a case report detailing an illness in female patients that was documented at the Regional General Banyumas Hospital, located in Central Java, Indonesia. This case report serves as the preliminary record of a bladder wall abscess that was identified prior to, during, and after transurethral resection of bladder mass. This case report of urological surgery aimed to elucidate an uncommon clinical phenomenon [4–6]. This case report is in line with the SCARE Criteria [7].

## Case presentation

**Fig. 1:**
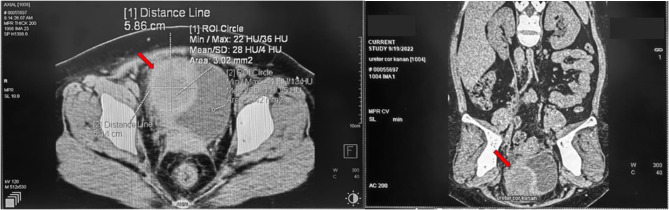
CT scan of the abdomen with contras demonstrating a bladder wall abscess ((a) red arrow) and a 5.86 × 5.14 cm delta 28 HU density in the right anteroinferior bladder wall

A female patient with normal BMI, 37 years of age, complained of chronic suprapubic pain and lower urinary tract symptoms (LUTS) for seven days. She had no entorological problems or gynecological problems or previous pelvic and urological surgeries. She was febrile on admission, with temperatures more than 38.5 °C. Blood and urine laboratory results were within normal limits. Computed tomography (CT) of the abdomen and pelvis revealed an inhomogeneous lesion in the right anterolateral bladder wall and other findings in both enterological and gynecological organs within normal limits. This lesion suggested a bladder tumour with differential diagnosis a bladder wall abscess caused by Hounsfield unit (HU) findings. Furthermore, computed tomography (CT) analysis revealed a tissue density of delta HU 28, measuring approximately 5.8 × 5.14 cm. (Fig. 1).

**Fig. 2:**
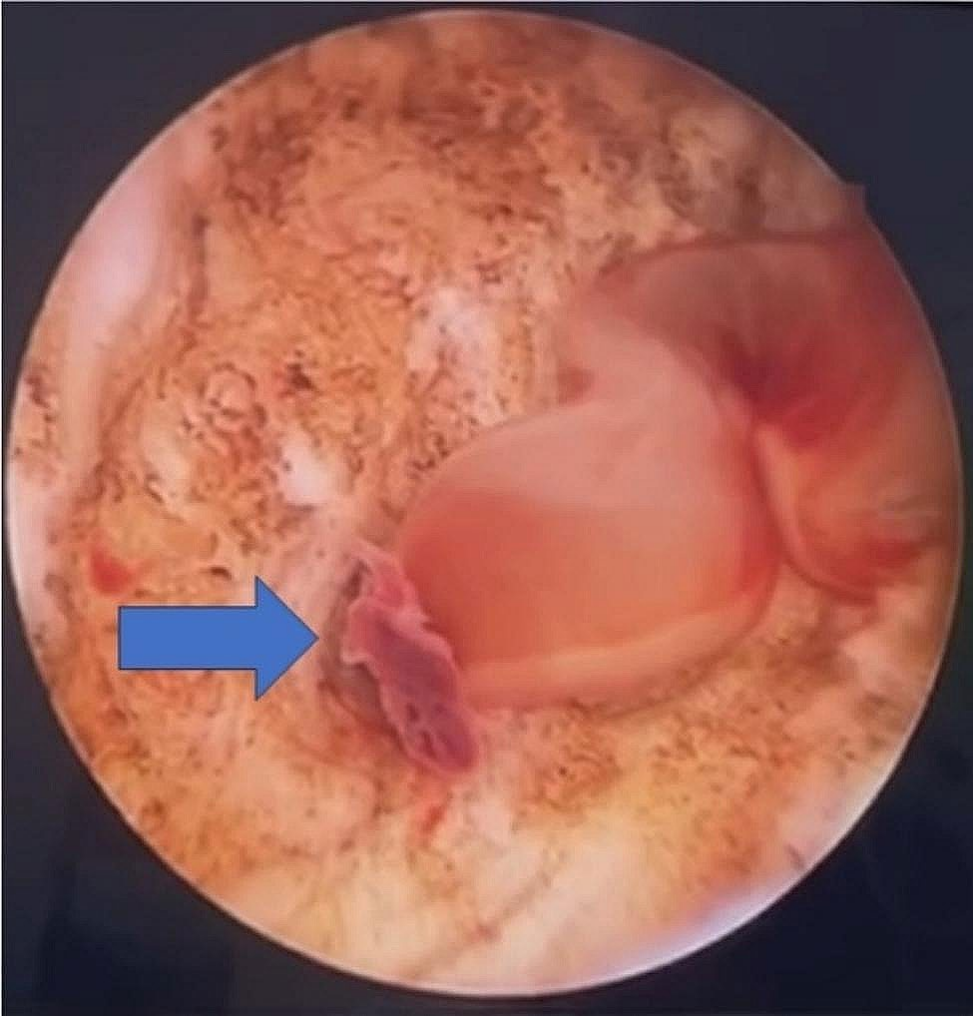
A resection was carried out on the bladder wall, and white-yellow purulent discharge came out. (Blue arrow)

The patient underwent cystoscopy, wall biopsy, and transurethral resection of a bladder tumour. Cephalosporin group II was administered as preoperative antibiotics and paracetamol was administered intravenously for pain and febrile release. The patient had no others comorbidities such as diabetes and hypertension, and no other medications, such as immunosuppression. She was a passive smoker. Her physical examination result were within normal limits. During Cystoscopy, we found an unidentified elevated lesion on the bladder wall, similar to a tumour, particularly at the base, right anterolateral to the urethra (Fig. 2). Transurethral resection of the mass was performed to obtain a specimen for pathological analysis.

**Fig. 3:**
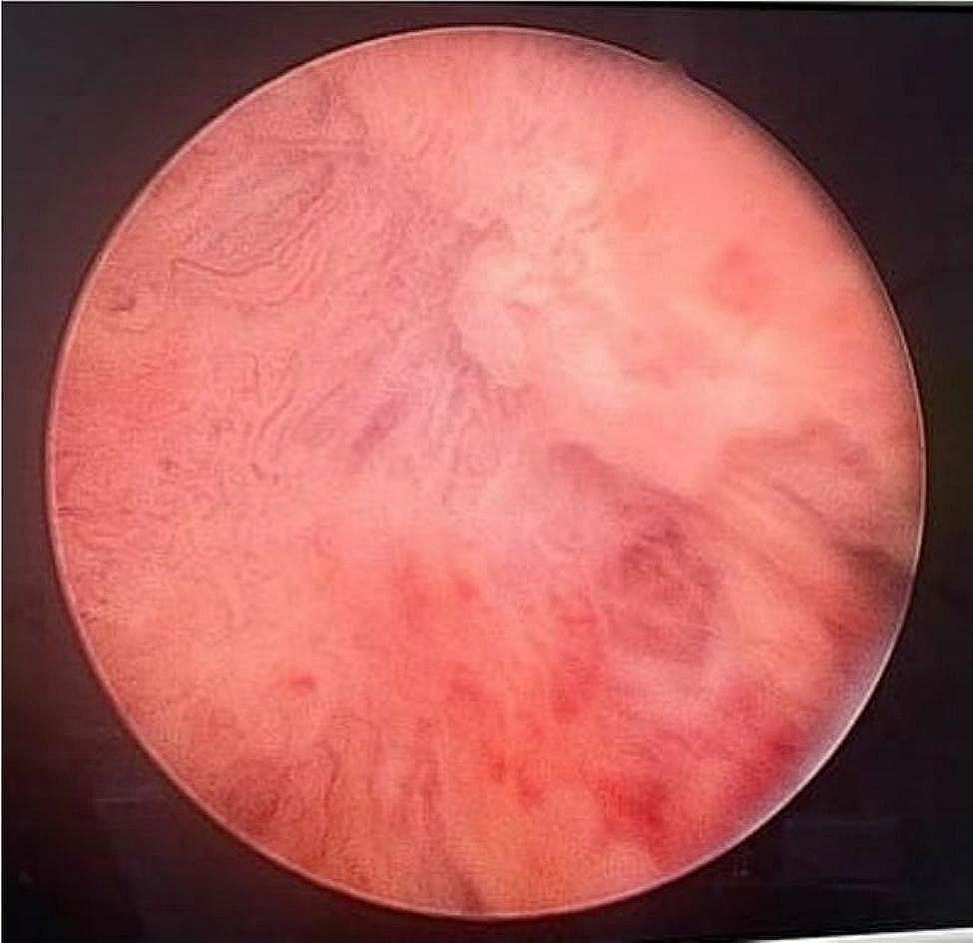
Base of bladder wall abscess

Purulent discharge (white-yellow colour) was accompanied by blood removal during transurethral resection of the entire bladder mass. All remaining exudate was removed until the bladder wall cavity was completely clean down to the base of the abscess (Fig. 3). Approximately 50 mL of purulent fluid was completely extracted and sent for culture. The tissue and base of the bladder abscess were subjected to a pathological analysis. After deroofing and evacuation of the abscess, we evaluated the bladder wall and obtanied good results without any complications such as bladder perforation. A 3-way Foley catheter with a 24-French orientation was inserted using saline irrigation. Irrigation and catheter removal were performed on the first day and third day, respectively. The patient was discharged from the hospital and administered the antibiotic cefixime for one week after the procedure. No complications were found during hospitalization.

Cultures on the lesion and urine did not grew up. On histological examination, inflammation of the urothelium and the tissue surrounding the abscess base did not indicate malignancy. The presence of small focal colonies with cellular characteristics on the surface of the urothelium was indicated by chronic cystitis with moderate proliferative activity and no papillary, in situ, or invasive malignancy. Two weeks after the procedure, the patient was followed up. She denied any symptoms of lower urinary tract or suprapubic abdominal discomfort. The patient was active and worked well.

## Discussion and conclusion

Bladder wall abscess is rare. Only three cases on adult patient that were documented [1–3]. A young man presented with significant dysuria and severe discomfort in the perineum [1], an elderly woman presented with a psoas abscess [2], and a 20-year-old male presented with a 2-week history of suprapubic pain, lethargy and intermittent fever [3]. In paediatric cases, spontaneous bladder wall abscesses are present in the neurogenic bladder [8, 9]. Bladder wall abscesses, among those that have been documented, exhibit a diverse array of clinical characteristics and presentations within healthcare facilities.

Our patient complained of suprapubic pain and LUTS, and had no prior urological, enterological and ginecological diseases. The observed variations could potentially be attributed to anatomical varitions between the urinary tracts of males and females, in addition to the patient’s age. The bladder lesion remained unidentified due to her lack of documented attendance at routine healthcare facilities. Recurrent urinary tract infections (UTIs) can induce abscess development. It is well established that recurrent infections induce changes in the epithelial cell wall during infancy. Furthermore, the immunosuppressive effects of medications that are not prescribed by a physician are a contributing factor. The patients consumed a glass of herb daily, consisting of steroids (dexamethasone) [10, 11]. Additionally, recurrent urinary tract infections have been documented in the patients’ medical histories. This indicates that individuals are afflicted with abscess development in the urinary tract.

The cause of bladder abscess is unknown [1]. Sepsis condition might be one of the possibility factors, although it could be spontaneous. Hematogenous spread might be contributed to the development of a bladder wall abscess during sepsis confitions [3]. In women with bladder wall abscesses, endoscopy intervention with cystoscopy might be a safe procedure with minimal complication and got the patient satisfaction [12]. 

In conclusion, the pathophysiological mechanisms underlying the development of bladder wall abscess remain unclear. Bladder wall abscesses treated with incision, drainage, and antibiotic treatment yielded satisfactory results.

## Data Availability

The datasets used and/or analysed during the current study are available from the corresponding author on reasonable request.
